# Veterinary fixed-dose antibiotics in Africa: a need for evidence-based regulation

**DOI:** 10.1186/s13567-026-01797-9

**Published:** 2026-06-18

**Authors:** Dishon M. Muloi, Hildah Kirimi, Joyce Mwaura, Eugine L. Ibayi, Arshnee Moodley

**Affiliations:** 1 Health Program, International Livestock Research Institute, Nairobi, Kenya; 2 Department of Veterinary and Animal Sciences, Faculty of Health and Medical Sciences, University of Copenhagen, Frederiksberg C, Denmark

**Keywords:** Fixed-dose combination, antibiotics, antimicrobial resistance

## Abstract

Fixed-dose combination (FDC) antibiotics are commonly used in animal health; however, their use faces increasing scrutiny due to concerns about unproven efficacy, inappropriate dosing, safety and limited regulatory oversight. We reviewed veterinary drug registries from Kenya, Uganda, Tanzania, Rwanda and Zimbabwe, identifying 2339 registered products, of which 867 (37.1%) contained antibiotics. Among these, 238 (27.5%) were FDCs, representing 45 unique combinations – most commonly two-antibiotic formulations such as penicillin/dihydrostreptomycin, tylosin/doxycycline and trimethoprim/sulfadiazine. Most formulations (77.8%, *n* = 35) contained two antibiotics, while 15.5% and 6.7% contained three and four antibiotics, respectively. Nearly half of FDC brands (47.1%, 112/238) contained antibiotics from both the Caution and Prudence categories, while 39.5% (94/238) contained antibiotics from the Prudence category alone. Notably, 8.4% (20/238) of FDC brands contained colistin, with or without enrofloxacin, and 5% (12/238) included vitamin combinations. Some antimicrobial FDCs may be clinically justified, for example, by broadening spectrum or achieving pharmacological synergy, but many lack a clear therapeutic rationale, underscoring the need for an evidence-based regulatory review and clearer criteria for appropriate veterinary FDCs.

## Introduction, methods and results

In many low- and middle-income countries (LMICs), antibiotics are easily accessible and widely used in the agri-food sector as cost-effective alternatives to more expensive and less accessible veterinary health interventions. Among these, fixed-dose combination (FDC) antibiotics – defined as formulations containing two or more active antimicrobial ingredients (AAIs) in a fixed ratio – represent a significant share of both human and veterinary antibiotic use [1]. While some FDCs are well established and clinically justified due to demonstrated synergistic or complementary effects such as amoxicillin–clavulanic acid and trimethoprim–sulfamethoxazole, public health and veterinary bodies have raised concerns about the proliferation of combinations lacking clear pharmacological or clinical rationale. Poorly justified FDCs may also pose safety risks, including additive or synergistic toxicity, and could potentially contribute to the emergence of antimicrobial resistance (AMR), particularly through the co-selection and amplification of multidrug-resistant organisms [2]. Despite these concerns, the manufacture, prescription and use of FDCs continue largely unabated, particularly in LMICs, where they are favoured for their perceived broad-spectrum efficacy and low cost, often in settings lacking diagnostic support [3]. While global debates have prompted regulatory action in human health, leading to bans or restrictions of FDC antibiotics in several countries, e.g., India [4], there has been comparatively little policy attention to the use of FDCs in animal health systems, particularly outside of high-income countries. Current data on the scale of FDC use in animal health remain limited, especially in African contexts. To address this gap, we analysed veterinary drug registries from five East and Southern African countries – Kenya, Uganda, Tanzania, Rwanda and Zimbabwe – to estimate the proportion of registered veterinary antibiotic FDCs and characterise the types of combinations approved for sale and use.

This study investigated the proportion of FDC antibiotics registered for veterinary use in five countries: Tanzania, Kenya, Uganda, Rwanda and Zimbabwe. Data on all registered veterinary products were systematically extracted from the websites of the respective national veterinary medicines authorities during an initial search conducted in November 2024. Each product was classified on the basis of its therapeutic indication. For products containing antibiotics, the AAIs were identified and recorded. FDC antibiotics were defined as formulations containing two or more AAIs, with or without additional non-antibiotic components such as vitamin supplements. The antibiotic FDCs were evaluated using the Antimicrobial Advice Ad Hoc Expert Group (AMEG) classification system from the European Medicines Agency (EMA), which categorises veterinary antibiotics into four groups based on their importance to human and animal health: Avoid, Restrict, Caution and Prudence [5]. Data were analysed descriptively to summarise the proportion and types of antibiotic FDCs registered in each country.

**Figure 1:**
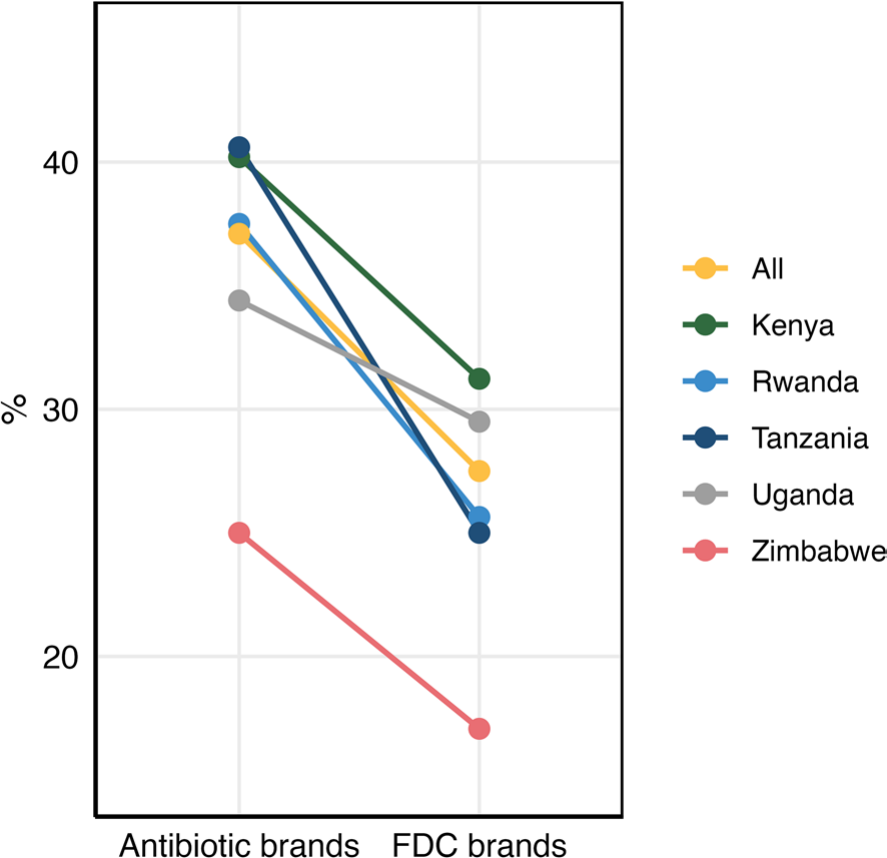
**Comparison of the pharmaceutical brands containing at least one antibiotic versus FDCs across five countries**

We identified 2339 unique veterinary pharmaceutical brands across the drug registries of Tanzania, Kenya, Uganda, Rwanda and Zimbabwe. Of these, 37.1% (*n* = 867) contained at least one antibiotic. Tanzania (40.6%, *n* = 258/635) and Kenya (40.2%, *n* = 349/868) had the highest proportions of antibiotic-containing products, while Zimbabwe had the lowest at 25.0% (*n* = 82/328). Among the antibiotic-containing brands, 27.5% (*n* = 238) were FDCs. Kenya (31.2%, *n* = 109/349) and Uganda (29.5%, *n* = 41/139) recorded the highest proportions, whereas Zimbabwe again had the lowest at 17.1% (14/82) (Figure 1).

**Figure 2:**
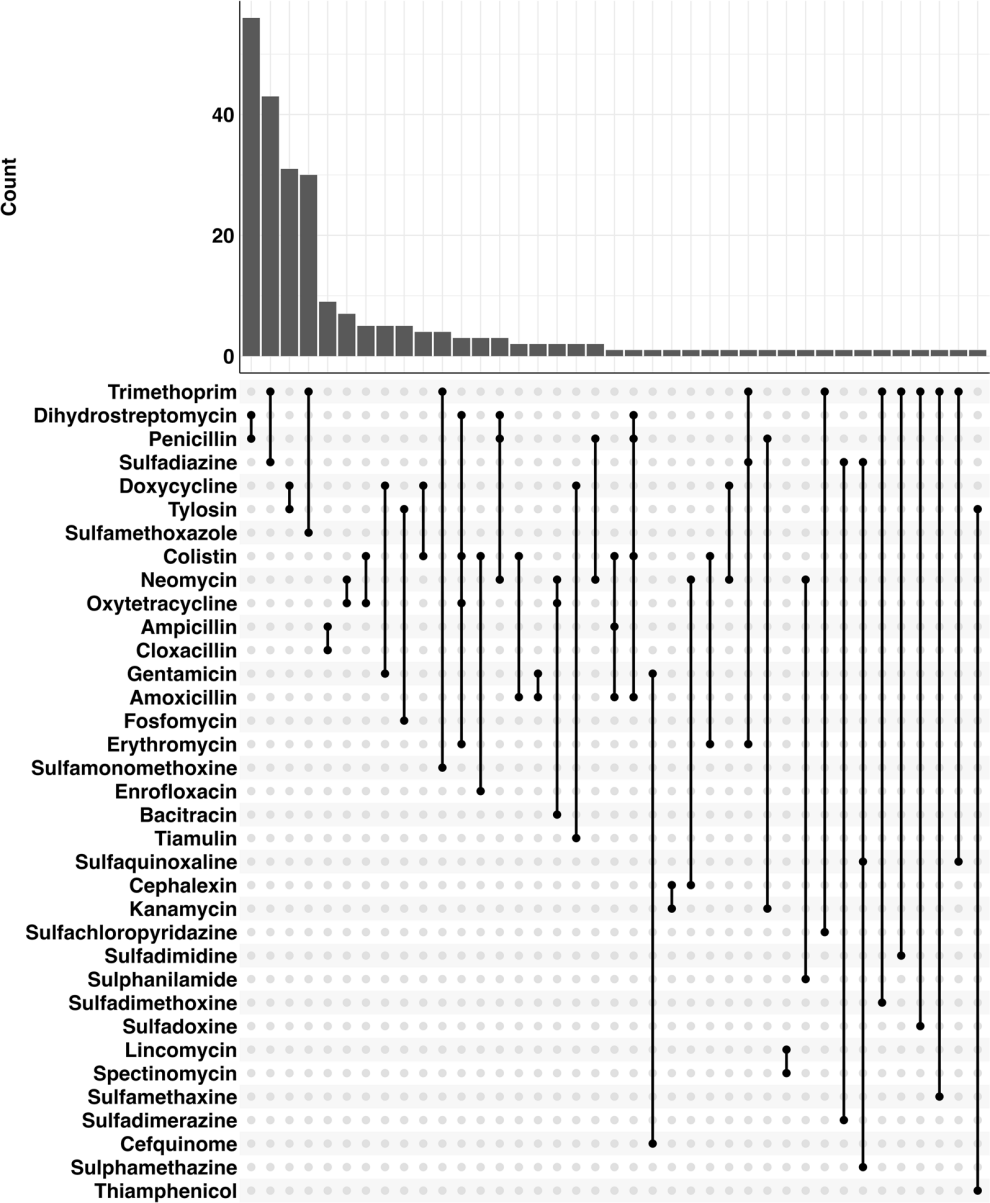
**Composition and frequency of antibiotic combinations in fixed-dose combination veterinary products**

We identified 45 distinct FDC formulations representing unique combinations of AAIs. Most formulations (77.8%, *n* = 35) contained two antibiotics and accounted for 95% (*n* = 226) of the 238 brands identified. Formulations containing three antibiotics represented 15.5% (*n* = 7) of FDCs and were present in 3.4% (*n* = 8) of brands, while those containing four antibiotics accounted for 6.7% (*n* = 3) of FDCs and were found in 1.6% (*n* = 4) of brands (Figure 2). The most common combinations across the 238 brands were penicillin + dihydrostreptomycin (23.5%, *n* = 56), tylosin + doxycycline (13.0%, *n* = 31) and trimethoprim + sulfadiazine (11.3%, *n* = 27).

Notably, 28.9% (*n* = 13) of the 45 unique formulations consisted of sulfonamide–trimethoprim combinations, represented across 140 of the 238 brands (58.8%).

**Figure 3:**
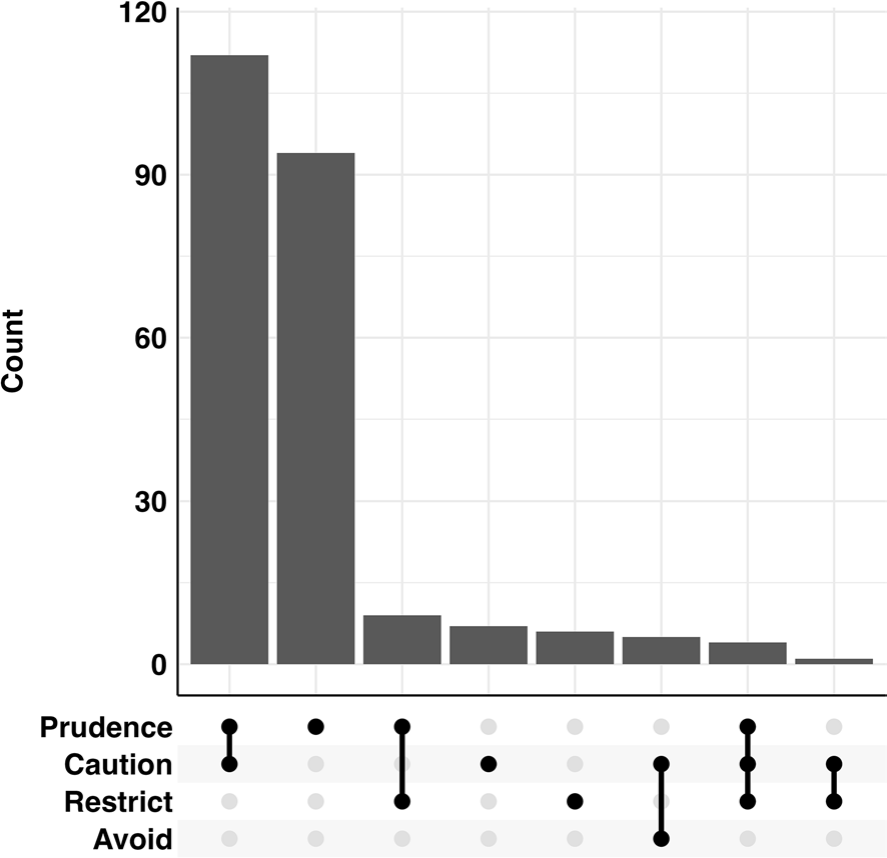
**Classification of antibiotic FDCs according to European Medicines Agency’s Antimicrobial Advice Ad Hoc Expert Group**

Additionally, 3.8% (33/867) of all antibiotic-containing brands and 5% (12/238) of FDC brands included vitamin supplements. Classification of the antibiotic FDCs based on the EMA’s categories revealed that most brands contained antibiotics from both the “Caution” and “Prudence” categories (47.1%, 112/238) or from the “Prudence” category alone (39.5%, 94/238). Notably, 8.4% (20/238) of FDC brands contained colistin; three of these also included enrofloxacin, both classified in the “Restrict” category. Five brands (2.1%) contained fosfomycin, an “Avoid”-category antibiotic (Figure 3).

## Discussion

Our study demonstrates that a significant proportion of veterinary antibiotics registered in five East and Southern African countries are FDCs, extending previous findings that were limited to individual countries or specific livestock production systems [6, 7]. Several FDCs we identified contained up to three or four antibiotics, including agents classified by EMA as “Restrict” or “Avoid”, and some also included additives such as vitamins. The continued sale and use of antibiotic FDCs in resource constrained settings, across both animal and human health, is driven by several interrelated factors: empirical treatment practices where bacteriological confirmation is impractical or deprioritised, aggressive marketing and sales promotion and weak regulatory oversight [4, 8]. These gaps can be translated into improvement opportunities: strengthening licensing policies for the manufacture (where present), importation and distribution of antibiotics, including banning certain FDCs; developing evidence-based national antibiotic formularies; regulating the promotion of inappropriate combinations; and improving diagnostic capacity to guide clinical decision-making. A more holistic analysis is also needed to understand why FDCs have become preferred prescribing choices in veterinary practice, how restrictions or bans could be effectively implemented and what the downstream impacts would be on antimicrobial consumption patterns and animal health outcomes. Similarly, there is also an urgent need to enhance pharmacovigilance systems at both national and regional levels, in parallel with efforts to harmonise regulatory policies for monitoring the use of FDCs and their associated adverse outcomes [9]. The establishment of the African Medicines Agency offers a timely and strategic platform to advance continent-wide regulatory oversight and stewardship of veterinary and human medicines [10].

A clear and internally accepted definition of what constitutes an “inappropriate” antibiotic FDC remains lacking – a major policy gap that limits effective regulatory action and undermines cohesive stewardship efforts. Some combinations identified in our study may nonetheless have clinical justification based on established pharmacological principles [11]. For example, approximately one third of the FDC formulations included a sulfonamide combined with trimethoprim. Sulfonamides are a large family of synthetic bacteriostatic antifolate agents, while trimethoprim inhibits dihydrofolate reductase; together they act synergistically by sequentially blocking the bacterial folate synthesis pathway, resulting in enhanced antimicrobial activity compared with either drug alone [12]. Most veterinary formulations use a fixed trimethoprim:sulfonamide ratio of about 1:5, adopted from human trimethoprim–sulfamethoxazole formulations, although emerging pharmacokinetic (PK)/pharmacodynamic (PD) studies suggest this ratio may not be optimal across animal species [13]. Despite these uncertainties, trimethoprim–sulfonamide combinations remain widely used in food-producing animals and are included in veterinary formularies in many countries [5, 14]. Another commonly identified combination was penicillin with dihydrostreptomycin. This pairing has historically been used to broaden antimicrobial coverage, targeting Gram-positive organisms through penicillin and Gram-negative bacteria through streptomycin, and has long been registered for veterinary use in livestock production across multiple countries. However, regulatory approaches vary, with some jurisdictions, such as  Australia, restricting or revoking its routine use in food-producing animals due to antimicrobial stewardship concerns [15]. In contrast, many of the other FDCs identified in this study appear to offer little clear therapeutic advantage and may instead promote indiscriminate “shotgun therapy”. Several products combined multiple antibiotics with overlapping spectra or unclear pharmacological rationale. For example, we identified formulations containing dihydrostreptomycin, oxytetracycline, colistin and erythromycin or penicillin, dihydrostreptomycin, amoxicillin and colistin within single products. Such multi-antibiotic “cocktails” raise significant concerns, as they simultaneously expose bacterial populations to several antimicrobial classes, potentially accelerating the selection and dissemination of multidrug-resistant organisms [16]. More broadly, the composition of many products suggests that formulations may have been developed without systematic evaluation of clinical efficacy, pharmacological compatibility or safety data [8]. It is important to strengthen regulatory system to manufacture appropriate FDCs for clinical treatment.

The combination of antibiotics with vitamins in some formulations has been reported in previous studies in several African countries such as Kenya [6, 17], Malawi [7] and Uganda [18]. The primary purpose of this practice remains unclear, and there is little evidence describing its justification, benefits or risks. We postulate that such combinations may reflect marketing strategies aimed at shaping consumer perceptions, creating the impression that these products function primarily as nutritional supplements rather than antimicrobials. This framing may obscure the presence of antibiotics and facilitate over-the-counter sales and unregulated use, particularly among farmers who may be unaware of the antimicrobial content. Under the EMA regulatory framework, antibiotic combinations with substances that do not enhance antimicrobial efficacy (e.g. vitamins) often fail to meet authorisation criteria [19]. However, comparable regulatory provisions are largely absent in the countries included in this study, allowing such products to remain available in veterinary drug markets.

While our study focused on five sub-Saharan African countries with accessible online veterinary registries, similar trends have been reported elsewhere in sub-Saharan Africa [20–22], highlighting the need for future multi-country investigations to quantify and characterise the use of FDCs more comprehensively [8]. We do not attempt to assess the clinical appropriateness of specific antibiotic FDCs in the focus countries, recognizing that such evaluations are complex, context-specific and beyond the scope of this study. Nonetheless, the lack of systematic evidence on the therapeutic justification, safety and effectiveness of many formulations represents an important research and regulatory gap that warrants urgent attention. It is possible that the registries may not capture all products on the market, or products might be registered but not widely sold.

In conclusion, a substantial proportion of veterinary antibiotics in East and Southern Africa are marketed as FDCs, some of which include critically important or last-resort antibiotics, and others that combine antibiotics with vitamins. Some combinations are clinically justified, for example, those designed to broaden antimicrobial spectrum or achieve pharmacological synergy and may offer clinical value when supported by evidence. However, many others appear to lack a clear pharmacological or therapeutic basis. The rationale for the manufacture and use of antimicrobial FDCs therefore warrants further investigation. Robust studies are needed to evaluate their efficacy, safety and potential contribution to the emergence and spread of antimicrobial resistance. Addressing this gap will require concerted efforts from the pharmaceutical industry, researchers and veterinary regulatory authorities to generate evidence, strengthen oversight of veterinary antimicrobial products and ensure that only combinations with clear therapeutic value remain in use.

## Data Availability

The datasets analysed during the current study are available in the websites of the respective national veterinary medicines authorities.

## Abbreviations

FDC: fixed-dose combination
AAIs: active antimicrobial ingredients
AMR: antimicrobial resistance
EMA: European Medicines Agency
AMEG: Antimicrobial Advice Ad Hoc Expert Group
